# Cigarette smoking might weaken the prognostic significance of cytochrome P450 2C19*2 polymorphism in acute myocardial infarction patients

**DOI:** 10.1111/jcmm.12797

**Published:** 2016-04-12

**Authors:** Mingyu Zhang, Xiaoxia Liu, Lei Wang, Yan Wang, Li Ju, Jianfei Li

**Affiliations:** ^1^ Department of CardiologyThe 4th Hospital of Harbin Medical UniversityHarbinHeilongjiang ProvinceChina; ^2^ Harbin Red Cross HospitalHarbinHeilongjiang ProvinceChina

**Keywords:** cytochrome P‐450 CYP2C19, genetic polymorphism, myocardial infarction, smoking, prognosis

## Abstract

Prognostic significance of cytochrome P450 2C19*2 polymorphism in acute myocardial infarction is still not well investigated. The aim of the study was to determine the relationship between the genetic polymorphism and the outcome of the acute myocardial infarction patients, and to further clarify the impact of smoking on such relationship. Six hundred acute myocardial infarction patients were enrolled. All of them provided blood samples and underwent clopidogrel treatment. The genetic polymorphism was determined by polymerase chain reaction–restriction fragment length polymorphism analysis, and the platelet function was assessed using conventional aggregometry. Of the included patients, 287 carried GG wild‐type genotypes, 225 carried GA genotypes and 88 carried AA genotypes. The platelet aggregation rate was significantly elevated in the AA genotype patients, mainly in the non‐smoking patients (*P* < 0.001) and the former‐smoking patients (*P* < 0.001). During 5‐year follow‐up period, after adjusted for multiple confounding factors, AA genotypes were associated with the increase in 5‐year mortalities in the non‐smoking patients [OR: 7.06, 95% confidence interval (CI): 2.16–11.49] and the former‐smoking patients (OR: 4.38, 95% CI: 1.05–9.40), but not in the current‐smoking patients (OR: 1.12, 95% CI: 0.60–2.31). In conclusion, the study suggested a potential role of P450 2C19*2 polymorphism as a prognostic indicator in acute myocardial infarction patients. We had also obtained some evidence that current smoking might weaken the prognostic significance of the genetic polymorphism in patients.

## Introduction

Acute myocardial infarction (AMI) is a common and life‐threatening clinical condition globally 1, 2. More than seven million persons suffer from the disease each year 3. Acute myocardial infarction usually occurs as a result of ruptured atherosclerotic plaque, platelet‐mediated thrombosis and prolonged myocardial ischaemia 4, 5. Antiplatelet drug clopidogrel is an appropriate and immediate treatment for AMI, and should be used for long‐term to prevent recurrent ischaemic events 6, 7.

However, some patients receive clopidogrel treatment, but still experience recurrent angina pectoris and AMI. Previous studies revealed significant interindividual variability in clopidogrel effect on platelet aggregation 8, 9, 10, which might inevitably lead to various clinical effectivenesses.

Clopidogrel is a prodrug, and requires multiple cytochrome (CYP) P450 isoenzymes for its activation, such as CYP1A2 and CYP2C19 11. Among these isoenzymes, the CYP2C19 is considered to be an extremely important enzyme in clopidogrel transformation 12. Hulot *et al*. reported that the CYP2C19*2 loss‐of‐function allele was associated with a marked decrease in platelet responsiveness to clopidogrel 13, and such single nucleotide polymorphism might partially explain the variability in clopidogrel effects. But another study showed no such relationship between the CYP2C19 genotype and the antiplatelet effect of clopidogrel 14. Thus, there was still insufficient evidence to determine the clinical impact of the CYP2C19*2 loss‐of‐function mutation.

Apart from genetic polymorphism, there are almost certainly other factors which can lead to the variability in clopidogrel effectiveness, such as percutaneous coronary intervention (PCI) and race 15, 16, 17, 18. Another compelling and unexpected factor is cigarette smoking. Smoking has the ability to increase the expression of the CYP1A2 19, which is one of the CYP P450 isoenzymes for clopidogrel activation. Bliden *et al*. proved that the clopidogrel treatment in current smokers was associated with the increased platelet inhibition and the lower aggregation compared with non‐smokers 20. Therefore, cigarette smoking seems to improve the clopidogrel effectiveness. Meanwhile, cigarette smoking is a major risk factor for cardiovascular disease, and is associated with a fivefold increased risk of acute ST‐segment elevation myocardial infarction 21. Thus, the impact of cigarette smoking on clopidogrel effectiveness and disease prognosis remains unclear.

In this study, we tried to reveal the relationship between the CYP2C19*2 genetic polymorphism and the outcome of the AMI patients who received clopidogrel treatment, and to further clarify the impact of cigarette smoking on such relationship.

## Materials and methods

### Participants

Between January 2008 and December 2011, a total of 600 patients with confirmed AMI in the Fourth Hospital of Harbin Medical University were included. Inclusion criteria were predefined as follows: (1) Ischaemic type chest pain lasting for more than 20 min., (2) Elevation of ST segment on electrocardiogram, and change in serial electrocardiogram tracing, (3) Rise of serum cardiac biomarkers 22, 23.

Smoking history in AMI patients must meet one of the following three conditions: (1) Current smoking: smoking more than 10 years (>10 cigarettes daily), and smoking within 4 weeks of AMI, (2) Former smoking: smoking more than 10 years (>10 cigarettes daily) and smoking cessation within 1 year of AMI, (3) Non‐smoking: never smoking in their lives.

The exclusion criteria were as follows: (1) Active haemorrhage or haemorrhagic tendency, such as peptic ulcer, haemorrhagic stroke and purpura, (2) Severe anaemia (Haemoglobin ≤ 70 g/l), (3) Liver dysfunction (Albumin < 30 g/l, Total bilirubin > 34.2 μmol/l, Alanine transaminase > 80 U/l, Aspartate transaminase > 80 U/l), (4) Renal impairment (Serum creatinine > 200 μmol/l), (5) Surgery or injury within the preceding 12 weeks, (6) Other serious diseases, such as tumour, infection, epilepsy, depression and mental disease, (7) Clopidogrel and aspirin intolerance, (8) Long‐term proton pump inhibitor usage, (9) Receive any antiplatelet or anticoagulant therapy in the last 4 weeks, (10) Smoking history did not meet any one of the conditions mentioned above.

Three hundred age‐ and gender‐matched healthy controls were randomly selected from the medical staff in the Fourth Hospital of Harbin Medical University. Written informed consent was obtained from all included patients and controls. This study was approved by the ethics committees of the Fourth Hospital of Harbin Medical University.

All patients received standard treatment. Each of them was treated with a dose of 300 mg clopidogrel and 300 mg aspirin before PCI. During the interventional procedure, at least one coronary stent was placed in the culprit vessel. To prevent recurrent ischaemic events, each patient was required to take clopidogrel (75 mg daily) and aspirin (100 mg daily) during the follow‐up period.

### Genetic polymorphism detection

Blood samples were obtained from all participants and were stored in tubes containing ethylene diamine tetraacetic acid. The genomic DNA was extracted using a commercial DNA isolation kit (Promega, WI, USA) according to the instruction manuals. The CYP2C19*2 polymorphism was detected by polymerase chain reaction (PCR) – restriction fragment length polymorphism (RFLP) analysis. The forward primer of CYP2C19*2 alleles was 5′‐ ACC AGA GCT TGG CAT ATT GTA TCT‐3′, and the reverse primer of such alleles was 5′‐GAT TCT TGG TGT TCT TTT ACT TTC T‐3′. Both primers were provided by BGI Beijing Corporation. The PCR was performed according to the standard procedures: (1) Initial denaturation at 94°C for 5 min., (2) 35 cycles of denaturation at 94°C for 30 sec., (3) Annealing at 60°C for 30 sec., (4) Elongation at 72°C for 30 sec.; (5) Final extension at 72°C for 10 min. The PCR‐amplified products were digested with SmaI restriction enzyme (Takara biotechnology, Dalian, China) and submitted for direct DNA sequencing.

### Platelet function assays

Each patient provided two blood samples for platelet function assay. One was obtained before clopidogrel administration. Another was obtained 72 hrs after clopidogrel administration and at least 24 hrs after termination of GP IIb/IIIa inhibitor treatment. All blood samples were stored in test tubes containing 3.8% sodium citrate, and were tested within 2 hrs.

The blood sample was centrifuged at 1000 r.p.m. for 10 min., and the resulting plasma was called as platelet‐rich plasma (PRP). After removing PRP, the sample was further centrifuged at 3000 r.p.m. for 5 min., and the resulting plasma was called as platelet‐poor plasma (PPP). The platelets were stimulated with 20 μmol/l adenosine diphosphate (ADP), and the platelet aggregation was assessed using a 4‐channel LBY‐NJ4 aggregometer (Precil, Beijing, China). The results were calculated according to the formula: (maximal light transmission of PRP/maximal light transmission of PPP) × 100%.

### Information collection

All patients and controls were interviewed by a single trained investigator, and the demographic data, smoking history and other necessary information were obtained in the conversations. The medical information were collected from medical records. The patients were followed up for 5 years (60 months) or until death. The primary outcome was death as a result of recurrent myocardial infarction. The secondary outcome was recurrent angina, non‐fatal AMI, stent thrombosis and cerebral stroke. The tertiary outcome was haemorrhage. BARC haemorrhage standard definition was used, and Bleeding Academic Research Consortium (BARC) type 1 to type 5 were all counted as haemorrhage outcome 24.

### Statistical analyses

All statistical analyses were performed performed with SPSS software, version 17.0 (SPSS, Inc., Chicago, IL, USA). Hardy–Weinberg genetic equilibrium test was used to estimate population representativeness. Statistical differences were assessed using independent sample *t*‐test in continuous variables and were tested using chi‐square test in categorical variables. *P* value < 0.05 was considered to be statistical significance. The odds ratio (OR) with 95% confidence interval (CI) was calculated using multivariable logistic regression analysis. If the 95% CI did not include ‘1′, the OR was considered statistically significant.

## Results

A total of 600 AMI patients and 300 health controls were included. All patients had completed 5‐year (60 months) follow‐up period or had been followed up until death. As shown in Table 1, the gender, age and ethnic group were equivalent between the patients and the controls (*P* = 0.254, *P* = 0.410 and *P* = 0.815). Compared with the controls, there were more patients with CYP2C19 681 mutant homozygotes (AA) (*P* < 0.001) and less patients with CYP2C19 681 wild‐type homozygotes (GG) (*P* = 0.004). In addition, the frequencies of the CYP2C19*2 genotypes obeyed the Hardy–Weinberg equilibrium in the patients (*P* = 0.331) and the health controls (*P* = 0.095).

**Table 1:** Characteristics of the participants in the study

	Patients	Controls	*P* value
No. of participants (*n*)	600	300	―
Gender, Male (*n*)	432	205	0.254
Age (years, mean ± S.D.)	62.1 ± 6.7	61.7 ± 6.9	0.410
Ethnic group, Han (*n*)	574	288	0.815
Smoking history			
Current smoking (*n*)	335	151	0.119
Former smoking (*n*)	106	68	0.073
Non‐smoking (*n*)	159	81	0.873
CYP2C19*2 genotype			
GG (*n*, %)	287 (47.8)	174 (58.0)	0.004
GA (*n*, %)	225 (37.5)	113 (37.7)	0.961
AA (*n*, %)	88 (14.7)	13 (4.3)	0.000

GG: CYP2C19 681 wild‐type homozygotes; GA: CYP2C19 681 mutant heterozygotes; AA: CYP2C19 681 mutant homozygotes.

**Figure 1:**
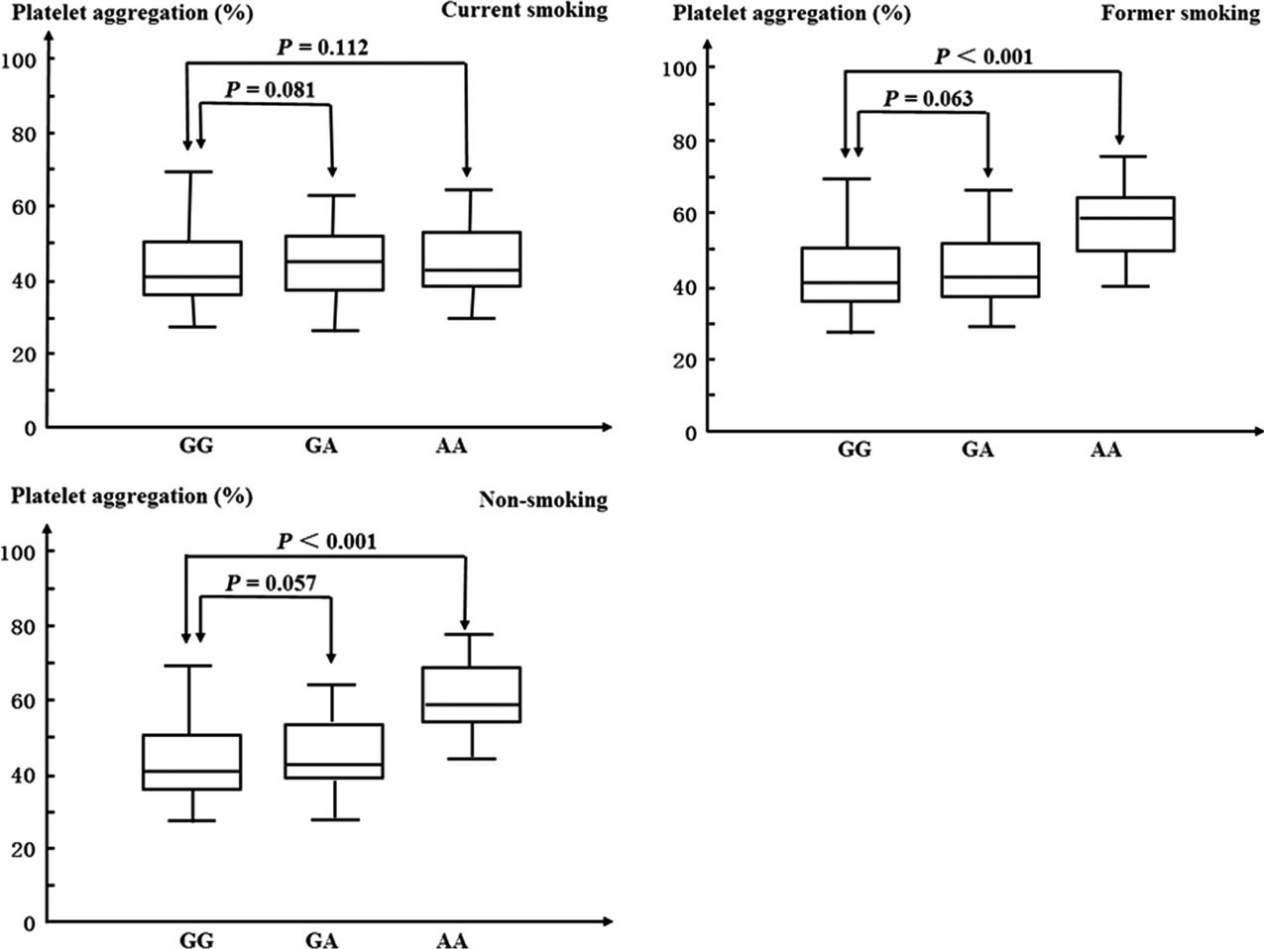
Adenosine diphosphate (concentration 20 μmol/l) induced platelet aggregation stratified by smoking history. GG: CYP2C19 681 wild‐type homozygotes, GA: CYP2C19 681 mutant heterozygotes. AA: CYP2C19 681 mutant homozygotes. The horizontal line within each box represents the median. The lower and upper borders of each box represent the 25th and the 75th percentiles. The vertical line extends from the minimum to the maximum value.

The patients were also stratified by smoking history, and were divided into three groups: current smoking group, former smoking group and non‐smoking group. The results of the platelet function assay were shown in Figure 1. In the non‐smoking group and the former smoking group, there were significant increased in ADP‐induced platelet aggregation percentage in the patients with AA genotypes compared with than in the patients with GG genotypes (GG: 42.3 ± 8.6%, AA: 54.1 ± 10.4%, *P* < 0.001 and GG: 40.1 ± 8.1%, AA: 52.9 ± 9.7%, *P* < 0.001). However, the difference between such two genotype groups was not observed in the current smoking patients (GG: 42.8 ± 8.9%, AA: 43.2 ± 8.3%, *P* = 0.112).

Of the included patients, 414 patients survived and 186 patients died during the follow‐up period. The demographic information, disease history and treatment history of the survival patients and the death patients were shown in Table 2. It was worth noting that a greater proportion of non‐smoking patients survived (*P* < 0.001), compared with the current smoking patients and the former smoking patients (*P* = 0.019 and *P* = 0.034).

**Table 2:** Characteristics of the patients in the survival group and the death group

	Survival	Death	*P* value
No. of participants (*n*, %)	414 (100.0)	186 (100.0)	―
Male (*n*, %)	302 (72.9)	130 (69.9)	0.441
Age (years, mean ± S.D.)	59.7 ± 5.8	67.3 ± 5.5	0.003
Han (*n*, %)	396 (95.7)	178 (95.7)	0.979
BMI (kg/m^2^, mean ± S.D.)	24.0 ± 1.2	26.9 ± 1.2	0.000
Hypertension (*n*, %)	201 (48.6)	129 (69.4)	0.000
Diabetes (*n*, %)	144 (34.8)	82 (44.1)	0.030
Hyperlipidaemia (*n*, %)	247 (59.7)	131 (70.4)	0.012
CVD (*n*, %)	73 (17.6)	58 (31.2)	0.000
Drinking (*n*, %)	62 (15.0)	48 (25.8)	0.002
Aspirin (*n*, %)	343 (82.9)	165 (88.7)	0.065
Beta‐blocker (*n*, %)	376 (90.8)	133 (71.5)	0.000
ACEI/ARB (*n*, %)	327 (79.0)	119 (64.0)	0.000
Statins (*n*, %)	352 (85.0)	147 (79.0)	0.070
Prior PCI (*n*, %)	34 (8.2)	47 (25.3)	0.000
Prior CABG (*n*, %)	21 (5.1)	35 (18.8)	0.000
Current smoking (*n*, %)	218 (52.7)	117 (62.9)	0.019
Former smoking (*n*, %)	64 (15.5)	42 (22.6)	0.034
Non‐smoking (*n*, %)	132 (31.9)	27 (14.5)	0.000

BMI: Body mass index; CVD: cerebrovascular disease; ACEI/ARB: Angiotensin‐converting enzyme inhibitor/Angiotensin receptor blocker; PCI: Percutaneous coronary intervention; CABG: Coronary artery bypass grafting.

As shown in Table 3, there were more survival patients with GG genotype and more death patients with AA genotype in the non‐smoking group (*P* < 0.001) and the former smoking group (*P* = 0.020). But in the current smoking group, GG and AA genotype distributions were similar between the survival patients and the death patients (*P* = 0.619).

**Table 3:** CYP2C19 681 genotypes of the patients in the survival group and the death group stratified by smoking history

Smoking history	Prognosis	Total (*n*)	Genotypes^a^			*P* value^b^	*P* value^c^
			GG (*n*)	GA (*n*)	AA (*n*)		
Current smoking	Survival	218	98	85	35	0.815	0.619
	Death	117	50	46	21		
Former smoking	Survival	64	42	18	4	0.168	0.020
	Death	42	19	15	8		
Non‐smoking	Survival	132	70	51	11	0.285	0.000
	Death	27	8	10	9		

a GG: CYP2C19 681 wild‐type homozygotes, GA: CYP2C19 681 mutant heterozygotes, AA: CYP2C19 681 mutant homozygotes.

b GG group *versus* GA group.

c GG group *versus* AA group.

During the follow‐up period, compared with the patients with GG genotype, more AA genotype patients in the non‐smoking and the former smoking groups suffered from adverse cardiovascular events, such as recurrent angina (OR: 4.63, 95% CI: 1.56–8.85 and OR: 3.66, 95% CI: 1.57–8.51), AMI (OR: 4.15, 95% CI: 1.16–7.24 and OR: 2.05, 95% CI: 1.09–5.03), stent thrombosis (OR: 5.17, 95% CI: 1.09–9.41 and OR: 3.79, 95% CI: 1.18–6.71) and cerebral stroke (OR: 4.44, 95% CI: 1.65–9.33 and OR: 3.39, 95% CI: 1.52–6.74). But in the current smoking group, the onset of the adverse cardiovascular events were similar between GG and AA genotype groups. The detailed results were shown in Table 4.

**Table 4:** Association between the CYP2C19 681 genotypes and the adverse cardiovascular events stratified by smoking history

	Multivariable adjusted OR (95% CI)^a^		
	Current smoking	Former smoking	Non‐smoking
Recurrent angina			
GG	Reference	Reference	Reference
GA	1.02 (0.49, 1.64)	1.54 (0.86, 4.57)	3.39 (0.64, 5.15)
AA	1.18 (0.79, 1.93)	3.66 (1.57, 8.51)	4.63 (1.56, 8.85)
Recurrent AMI			
GG	Reference	Reference	Reference
GA	1.27 (0.51, 3.51)	2.00 (0.31, 4.73)	3.17 (0.65, 5.24)
AA	1.30 (0.69, 2.72)	2.05 (1.09, 5.03)	4.15 (1.16, 7.24)
Stent thrombosis			
GG	Reference	Reference	Reference
GA	0.71 (0.43, 1.59)	2.97 (0.32, 6.48)	4.97 (0.89, 7.73)
AA	0.95 (0.68, 1.93)	3.79 (1.18, 6.71)	5.17 (1.09, 9.41)
Cerebral stroke			
GG	Reference	Reference	Reference
GA	0.82 (0.47, 1.71)	3.16 (0.78, 5.29)	2.98 (0.93, 5.88)
AA	0.76 (0.48, 1.85)	3.39 (1.52, 6.74)	4.44 (1.65, 9.33)

a OR (95% CI): Odds ratio (95% confidence interval). Multivariable adjusted OR were adjusted for age, body mass index, onset of hypertension, diabetes, hyperlipidaemia, cerebrovascular disease, drinking, beta‐blocker usage, angiotensin‐converting enzyme inhibitor usage, angiotensin receptor blocker usage, prior percutaneous coronary intervention and prior coronary artery bypass grafting.

CI: confidence interval; GG: CYP2C19 681 wild‐type homozygotes; GA: CYP2C19 681 mutant heterozygotes; AA: CYP2C19 681 mutant homozygotes; AMI: Aute myocardial infarction.

In the non‐smoking and the former smoking groups, after adjusted for multiple confounding factors, the 5‐year mortalities of the AA genotype patients increased compared with the GG genotype patients (OR: 7.06, 95% CI: 2.16–11.49 and OR: 4.38, 95% CI: 1.05–9.40). But no such change was found in the current smoking group (OR: 1.12, 95% CI: 0.60–2.31). The patients were also divided into two subgroups according to the haemorrhage history during the follow‐up period. The results did not change, which revealed that the impact of smoking on the prognosis was independent of the haemorrhage history. The detailed results were shown in Table 5.

**Table 5:** Association between the CYP2C19 681 genotypes and the death during the follow‐up period stratified by smoking history

	Multivariable adjusted OR (95% CI)^a^		
	Current smoking	Former smoking	Non‐smoking
Total			
GG	Reference	Reference	Reference
GA	1.05 (0.61, 1.69)	1.79 (0.68, 4.22)	1.52 (0.53, 4.27)
AA	1.12 (0.60, 2.31)	4.38 (1.05, 9.40)	5.06 (2.16, 11.49)
Haemorrhage			
GG	Reference	Reference	Reference
GA	1.12 (0.52, 1.77)	1.67 (0.59, 4.51)	1.12 (0.87, 3.87)
AA	1.09 (0.48, 2.17)	4.21 (1.14, 8.37)	5.79 (1.91, 7.33)
Non‐haemorrhage			
GG	Reference	Reference	Reference
GA	1.21 (0.39, 1.94)	1.72 (0.61, 4.18)	1.33 (0.51, 4.80)
AA	1.16 (0.53, 2.11)	4.00 (1.01, 9.03)	6.88 (2.00, 11.16)

a OR (95% CI): Odds ratio (95% confidence interval). Multivariable adjusted OR were adjusted for age, body mass index, onset of hypertension, diabetes, hyperlipidaemia, cerebrovascular disease, drinking, beta‐blocker usage, angiotensin‐converting enzyme inhibitor usage, angiotensin receptor blocker usage, prior percutaneous coronary intervention and prior coronary artery bypass grafting.

CI: confidence interval; GG: CYP2C19 681 wild‐type homozygotes; GA: CYP2C19 681 mutant heterozygotes; AA: CYP2C19 681 mutant homozygotes.

## Discussion

In the study, we enrolled 600 AMI patients and 300 health controls, completed 5 years follow‐up period, and tried to reveal the association between the onset of the CYP2C19 681 mutant genotypes and the poor prognosis in AMI patients. Several evidence supporting the hypothesis had been found mainly in the non‐smoking and the smoking cessation population. First, there were more CYP2C19 681 gene mutation (AA genotype) in AMI patients than in controls, which showed the potential relationship between CYP2C19 681 genotype and the onset of AMI. Second, when the patients were treated with antiplatelet drug clopidogrel, the platelets showed better aggregation function in the AA genotype patients. Third, the patients with AA genotypes were more prone to adverse cardiovascular events during the follow‐up period. Fourth, after excluding the effects of multiple confounding factors, 5‐year survival incidence was significantly higher in the patients without mutant genotypes compared with the patients with AA genotypes. Therefore, based on the results in the study, the CYP2C19*2 polymorphism might be a potential marker for the prognosis in AMI patients.

However, most positive results mentioned above were not observed in the current smoking patients in our study. Possible mechanisms were as follows: (1) The CYP1A2 was another important metabolic enzyme, which could promote the activation of clopidogrel, (2) Cigarette smoking was able to elevate the expression of the CYP1A2 and to improve the efficacy of clopidogrel, (3) This might partly counteract the effect of the CYP2C19 681 gene mutation. Years ago, Bliden *et al*. conducted a study suggesting that clopidogrel therapy in current smokers was associated with increased platelet inhibition and lower aggregation 20. This research was consistent with our study. Therefore, cigarette smoking might weaken the prognostic significance of the CYP2C19 in AMI patients. However, further study should be conducted to explore the detailed mechanisms.

Based on our results, cigarette smoking might partly increase the efficacy of clopidogrel. But this did not mean that smoking could improve the prognosis of AMI patients. As we all know, long‐term smoking could seriously damage blood vessels, and lead to vascular stenosis and heart attack 25, 26, 27. At least half of long‐term smokers died earlier as a result of smoking 28, 29. Therefore, the efficacy of clopidogrel apparently could not compensate for the systemic damage caused by long‐term smoking. Our study achieved consistent results. As shown in Table 2, a greater proportion of non‐smoking patients survived during the follow‐up period, compared with the current smoking patients. Undoubtedly, Smoking was extremely harmful to health, and was related with poor prognosis in cardiovascular disease patients.

In the study, the patients were divided into three smoking history groups including the former smoking group. Previous study suggested that the time course of CYP1A2 activity changes after smoking cessation in heavy smokers was not entirely clear 30. To avoid bias, ‘the former smoking’ was defined as ‘smoking more than 10 years (>10 cigarettes daily), and smoking cessation within 1 year of AMI’.

Mature and reliable technology was adopted in the study. The CYP2C19*2 polymorphism was detected by PCR–RFLP analysis, and the platelet function was assessed using conventional aggregometry. A great many of previous studies focused on the relationship between CYP2C19 polymorphism and clopidogrel therapy – related diseases also adopted these technologies, and reported comparable results 31, 32. These might support the validity of our detection on the CYP2C19*2 polymorphism and the platelet function.

There were several limitations in the study. On the one hand, we did not study other CYP P450 isoenzymes and their genetic polymorphism, particularly CYP1A2, which affected the metabolism of clopidogrel. On the other hand, our study was not a randomized controlled trial, and could not rule out the influence of selection bias. Therefore, some randomized controlled studies which included a variety of clopidogrel‐related metabolic enzymes should be conducted.

In conclusion, our study suggested a potential role of CYP2C19*2 polymorphism as a prognostic indicator in AMI patients. We had also obtained some interesting evidence that cigarette smoking might weaken the prognostic significance of the genetic polymorphism in patients. In addition, though cigarette smoking might improve the efficiency of clopidogrel, it was still related with poor prognosis in cardiovascular disease patients and was extremely harmful to health.

## Conflicts of interest

The authors confirm that there are no conflicts of interest.
